# Local alignment of two-base encoded DNA sequence

**DOI:** 10.1186/1471-2105-10-175

**Published:** 2009-06-09

**Authors:** Nils Homer, Barry Merriman, Stanley F Nelson

**Affiliations:** 1 Department of Computer Science, University of California Los Angeles, Los Angeles, California 90095, USA; 2 Department of Human Genetics, David Geffen School of Medicine, University of California Los Angeles, Los Angeles, California 90095, USA

## Abstract

**Background:**

DNA sequence comparison is based on optimal local alignment of two sequences using a similarity score. However, some new DNA sequencing technologies do not directly measure the base sequence, but rather an encoded form, such as the two-base encoding considered here. In order to compare such data to a reference sequence, the data must be decoded into sequence. The decoding is deterministic, but the possibility of measurement errors requires searching among all possible error modes and resulting alignments to achieve an optimal balance of fewer errors versus greater sequence similarity.

**Results:**

We present an extension of the standard dynamic programming method for local alignment, which simultaneously decodes the data and performs the alignment, maximizing a similarity score based on a weighted combination of errors and edits, and allowing an affine gap penalty. We also present simulations that demonstrate the performance characteristics of our two base encoded alignment method and contrast those with standard DNA sequence alignment under the same conditions.

**Conclusion:**

The new local alignment algorithm for two-base encoded data has substantial power to properly detect and correct measurement errors while identifying underlying sequence variants, and facilitating genome re-sequencing efforts based on this form of sequence data.

## Background

DNA sequence comparison is a common problem in biology. In this problem, we wish to measure the similarity of two sequences of DNA. Hamming distance [1] can be used to quantify similarity but forces the two sequences to be of the same length. More generally, the idea of a weighted edit distance can be applied, which allows for base changes, insertions and deletions [2], with weights chosen to reflect their likelihood of occurrence. Given some set of operators that can modify a sequence, we wish to find the set of edit operators that transforms one sequence into a (sub)sequence of the other by maximizing a similarity score. This problem can be solved by a dynamic programming algorithm, which was first described in 1970 [3]. This led to the Smith-Waterman algorithm [4] that has been a critical component of local sequence alignment. Affine gap penalties were subsequently introduced, whereby in practice the per-base average penalty decreases, but the overall penalty increases with longer length[5]. This algorithm has a known O(*nm*) running time and O(min(*n*, *m*)) space requirements, for both finding a maximum similarity score and finding a transformation that achieves the maximum similarity score, where *n *and *m *are the lengths of the two sequences to be compared [3-9]. The resulting algorithm has become the standard for DNA sequence comparison [3,4,10,11].

**Figure 1:**
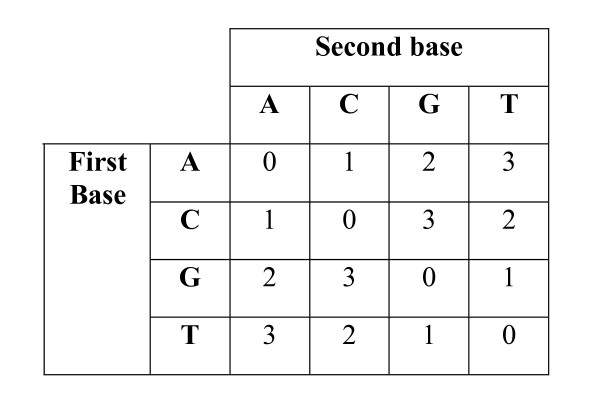
**The function Φ**. Φ is a function that encodes two bases as a color. Each color is represented by a number ∈ {0, 1, 2, 3}.

Sequence comparison has an important application to re-sequencing, whereby a DNA sequence that is observed may differ from a reference due to biological events or measurement errors. We wish to find the maximum similarity score between the observed sequence and a substring of the reference sequence. This is referred to as local sequence alignment and is typically a final finishing step in a two-stage search process found in many current sequence alignment tools [12-15] (Homer N, Merriman B, Nelson SF: BFAST: the BLAT-like Fast Accurate Search Tool for Large-Scale Genome Resequencing, submitted) that support alignment of a short sequence to an entire genome. Among the 'next-generation' DNA sequencing technologies that produce millions to billions of short sequence reads, there is one (the SOLiD™ platform [16-18]) that does not observe each DNA base (A, C, G, or T) individually, but measures successive sequential pairs, with the 16 possibilities encoded degenerately in groups of four, using four "color" codes (see Figure 1 for details). The resulting two-base encoded form of data is referred to as color space sequence data, to distinguish this from the decoded base space sequence data[16,17]. For example, a 50-base DNA sequence would be encoded as 49 sequential two-base measurements, each of which is one of four states (colors). Given the first base of the sequence as a boundary condition (which in practice is the known last base of the sequencing primer), the chosen encoding allows for the bases to be sequentially decoded, moving from first to last, in a fully deterministic manner. While the actual two-base encoding used has a number of interesting and useful algebraic properties [17], the most important properties are that a single base change to the DNA base sequence results in two adjacent color changes in the color space sequence, and that an isolated error in color space will cause all subsequent bases to be altered in the decoding. The result is that isolated measurement errors and real variants have distinguishable signatures that in principle provide some ability to perform error detection and correction. In particular, two specific adjacent measurement errors are required to produce a single base change error in the decoded sequence, so that the base calling error rate could be reduced to the square of the intrinsic measurement error rate (which is ~1%–10%), if the encoding properties can be fully exploited when comparing the color space reads to a reference DNA sequence.

In a typical re-sequencing experiment using next-generation sequencing technology, millions of short sequence "reads", 20–100 bases in length, must be aligned to a large reference genome, such as the human genome. This demands an initial search space reduction step [12-14,18-20] (Homer N, Merriman B, Nelson SF: BFAST: the BLAT-like Fast Accurate Search Tool for Large-Scale Genome Resequencing, submitted) prior to performing the more expensive optimal local alignment. This first step typically involves some form of indexed look-up or hashing of the full genome or reads, so that a small number of candidate alignment locations are quickly obtained for each read, in a way that is tolerant of the read containing errors or real variants relative to the reference. The optimal local alignments are then used to select which of these candidates is the true location, as well as to identify the differences from the reference sequence at that location. In the case of color space data, the look-up phase can be performed entirely in color space, using the color-space encoded form of the reference genome to find candidate locations for each color space read. The optimal alignment algorithm described here would then be used as the finishing step, which simultaneously decodes, identifies color (measurement) errors, and optimally aligns resulting DNA sequence to a short candidate segment of the reference sequence, typically 100–1000 bases in length (to allow for insertions and deletions in the read).

## Results

### Power of two-base encoding

**Figure 2:**
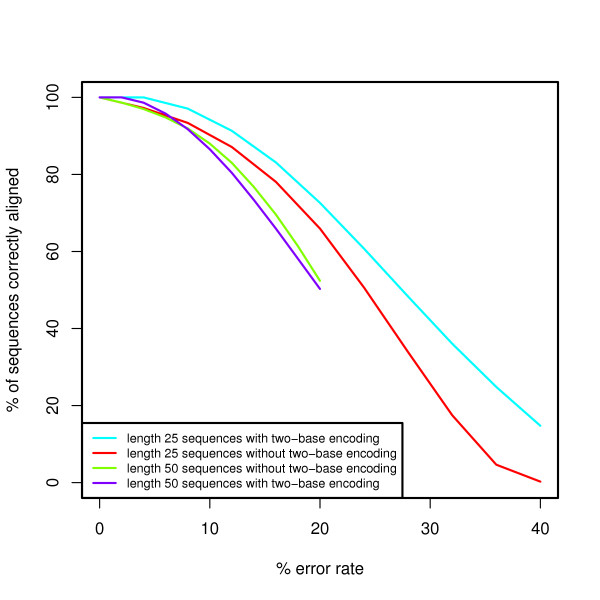
**Power evaluation for sequences with errors**. We assess the power to align sequences with and without two-base encoding in the presence of a per-base or per-color error rate respectively.

**Figure 3:**
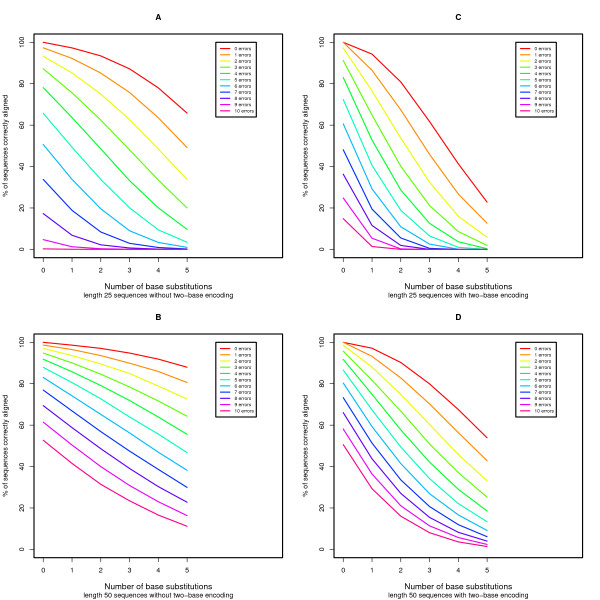
**Power evaluation for sequences with errors and base substitutions**. We assess the power to align sequences with and without two-base encoding in the presence of errors and base substitutions.

**Figure 4:**
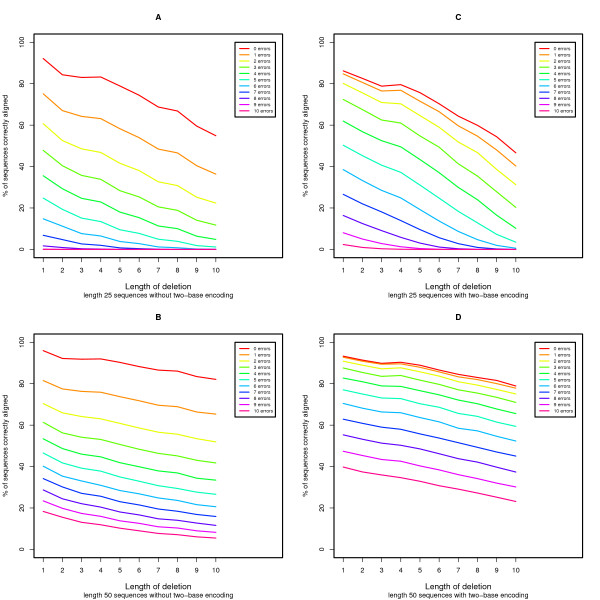
**Power evaluation for sequences with errors and a contiguous deletion**. We assess the power to align sequences with and without two-base encoding in the presence of errors and a contiguous deletion.

**Figure 5:**
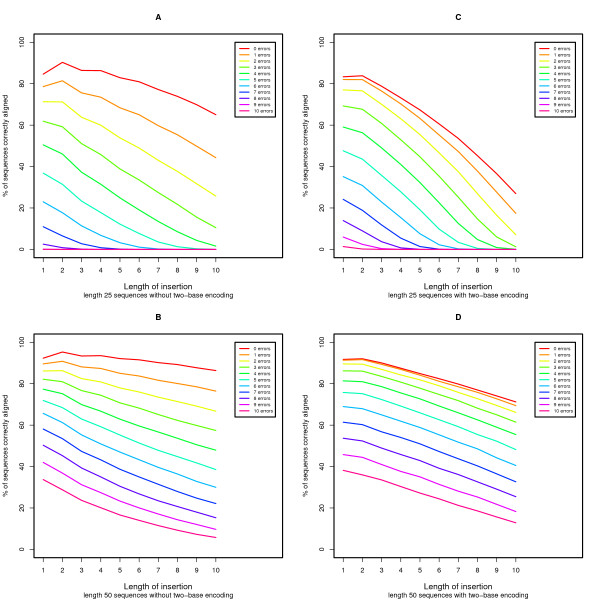
**Power evaluation for sequences with errors and a contiguous insertion**. We assess the power to align sequences with and without two-base encoding in the presence of errors and a contiguous insertion.

We performed simulations to evaluate the power of our proposed algorithm to align sequences with two-base encoding compared to the local alignment without two-base encoding (see Methods for details). We model errors as base substitutions when the sequence is not encoded and model errors as color substitutions (encoding errors) when the sequence is encoded in color space. In Figure 2, we demonstrate that for sequences with increasing error rates, aligning with two-base encoding is nearly equal to (for longer reads) or more powerful than (for shorter reads) aligning without two-base encoding. Nevertheless, if we examine base substitutions in the presence of error (Figure 3), the current algorithm is unable to properly align sequences with an increasing number of base substitutions in the presence of a small number of random errors. The scenario where there are many base substitutions that are not errors (in this case Single Nucleotide Polymorphisms or SNPs) is rare, especially in the human genome[21,22], and therefore this behavior is tolerable. In Figures 4 and 5 we see the power to detect deletions and insertions with an increasing number of errors. For a contiguous deletion the power to align such sequences is equal or greater with two-base encoding, except in the case of a one base deletion with no errors where the power is slightly reduced. For a contiguous insertion, the case is more ambiguous. As expected with greater error (≥ 5 errors), the two-base encoding becomes more powerful. Nevertheless, for a small amount of error, the two-base encoding has lower power to align longer contiguous insertions. In this case, over-correction can occur, whereby we align with too many color substitutions rather than the contiguous insertion. This may be mediated by decreasing the penalty for extending an insertion or deletion, although this may reduce the accuracy for high-error sequences without insertions or deletions.

### Performance of two-base encoding

We performed simulations to evaluate the performance of the current algorithm compared to the local alignment without two-base encoding (see Methods for details). We found that for length 25 and 50 color space sequences our algorithm was 36 and 28 times slower, respectively, than the standard Dynamic Programming algorithm applied to base space sequence. Although the algorithmic complexity as a function of read length and reference length is not increased, the absolute number of operations does increase (see Methods), and thus we observe a decrease in the speed performance compared to sequences without the two-base encoding. This performance decrease is particularly relevant given that an experimentalist may be required to choose between competing sequencing technologies that do not utilize the two-base encoding scheme and sequencing technologies that do use the two-base encoding scheme. Two base encoding has potentially powerful error correction modes and at the time of this publication is able to generate substantially more data than direct sequencing approaches. Thus, the two base encoding strategy while preferable in some scenarios for base error correction and better performance of alignment does impose a need for increased computational capacity largely due to the local sequence alignment complexity.

## Discussion

Although the power of this algorithm enables accurate alignment of color space sequences with greater error, it is also computationally an order of magnitude more expensive than the standard dynamic programming algorithm applied in sequence space. To partially mitigate this, the performance can be optimized without changing the results by employing some simple search space reduction and greedy search techniques, as follows: first, decode the encoded sequence by the standard deterministic rules and perform an exact string matching search. If an exact match is found, then the algorithm stops. Upon unsuccessful return, we find a lower bound for the optimal similarity for the proposed algorithm by first performing our two-base encoded alignment but without allowing insertion or deletion edits, which substantially reduces the computational cost. Using this lower bound, we then reduce the search space of our full algorithm by omitting the paths where the search parameters that permit detection of insertions or deletions would result in a score below the established lower bound. In this manner, the empirical running time of the algorithm can be improved by approximately 20% (data not shown) while still obtaining the true optimal alignment.

We note that the general strategy of two-base encoding in color space is possible to apply in more complex formats for error correction. For instance, three or more bases may be encoded by four or more colors. This would further increase the power of discriminating between encoding errors and base substitutions, albeit at a substantial added cost in local alignment performance. In practice these alternate encodings could further reduce false-positives detections when the goal is to find biological variants with next-generation sequencing technology with relatively high measurement error rates. This may be an advantageous strategy, for example, to increase read lengths by accepting noisier color space reads that are correctable after alignment. The current algorithm can be extended to accommodate these generalizations, and in future work we will investigate the detailed performance properties of such hypothetical encodings.

The present algorithm can be readily extended to include support for the case where sequence data is missing or unavailable, in either the given color-encoded sequence or in the target base space sequence. We introduce a fifth color code to represent an unknown color in encoded sequence, and a fifth base code (traditionally "N") to represent an unknown base in the decoded or target sequence. To incorporate an unknown encoding color we modify the color substitution function Π to include a score for this fifth unknown color and any other color. To incorporate an unknown base in the target, we modify the base substitution function Δ to include a score for the unknown base and any other base. Also a simple modification to the initialization step in the algorithm is required if the start base *p *is not known. While we do not rely on quality values for each color read, however it is possible to incorporate into the current alignment algorithm quality values that represent the certainty of color calling similar to sequence calling with Phred scores [23-26] by weighting the color substitution function Π.

Finally, Figures 2, 3, 4, and 5 demonstrate the power to correctly align two-base encoded sequences in the presence of a large number of color errors. Depending on the distribution of sequences with a given number of errors, two-base encoding and this algorithm may make it feasible to accept higher error sequences generated by next-generation sequencing technology, improving both throughput and cost-effectiveness. Additionally, we place a constraint on our scoring functions, making a conscious choice to prefer a base substitution to two adjacent color substitutions that would cause that base to match the reference. This is by no means the only constraint available, but serves to help define the trade-off in power to detect errors over biological variants. In these practically important but ambiguous cases, a decision must be made over which scenario to prefer, and in practice this ambiguity can be overcome by using coverage where multiple sequences observe the same event.

## Conclusion

DNA sequence alignment algorithms have been thoroughly studied in molecular biology, resulting in well-developed Dynamic Programming algorithms that optimize an edit distance to find optimal alignments between two sequences. However, there is a resurgence of interest in sequence alignment due to large scale re-sequencing efforts made possible by massively parallel sequencing technology. The classical algorithm remains an ideal approach for local alignment of such short-read sequence data, but some sequencing technologies produce reads in encoded form, which must be decoded to obtain standard DNA sequence. We extend the previous class of Dynamic Programming algorithms to allow for errors in the encoding, as well as the usual base substitutions, insertions and deletions. Our algorithm remains O(*nm*) time, where *n *and *m *are the length of the encoded and target sequence respectively. We show in practice that performance is decreased due to the added complexity of considering encoding errors, although this can be somewhat mitigated by standard search optimization. This performance decrease must be kept in mind when comparing the overall computational cost of analyzing various next-generation sequencing technologies. Using this new algorithm, local sequence alignment as well as error detection and correction are performed in a reliable and systematic manner, enabling the direct comparison of encoded DNA sequence reads to a candidate reference DNA sequence. This new algorithm should facilitate the use of two-base encoded data for large-scale re-sequencing projects.

## Methods

### The Problem

To solve the DNA sequence comparison problem for encoded sequences, we follow a constructive approach. Given an encoded DNA sequence *c *= *c*_1_,..., *c*_*n*_, we wish to maximize the similarity between *c *and some regular DNA sequence *y *= *y*_1_,..., *y*_*m*_, with the valid edit operators Σ. In this case the alphabet is {*A, C, G, T*} corresponding to the bases in DNA, and the encoded alphabet is {*0, 1, 2, 3*}. We assume the encoded sequence is composed of a two base encoding, referred to as colors, as well as assume a known start base *p*, which is known in practice [16,17,27]. The valid edit operators are:

1. A base substitution, which substitutes one base for another in the encoded sequence after decoding.

2. An insertion, which inserts a base into the encoded sequence after decoding.

3. A deletion, which deletes a base from the encoded sequence after decoding.

4. A color substitution, which substitutes one encoded color for another.

**Figure 6:**
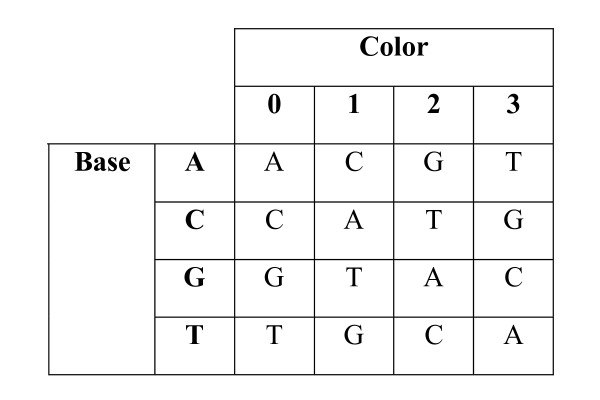
**The function Γ**. Γ is a function that encodes one base and one color as a base.

Operators 1–3 can be applied to base sequence and therefore we assume that all color substitutions are applied to the encoded sequence, then the sequence is decoded to allow the application of operators 1–3. We assign scores to each operator. The function Δ (*B*_1_, *B*_2_) that returns the base substitution score for substituting base *B*_2 _for base *B*_1_. The score ρ is applied for the first insertion or deletion operator used. Any insertion or deletion operator that is applied so that the insertion or deletion is extended has a score *ε*. Therefore, for a length *g*>0 base insertion or deletion, the cost of the entire insertion or deletion is *ρ *+ *ε *(*g*-1) and has an average per-gap cost of (*ρ *+ *ε *(*g*-1))/*g*. In practice, this affine gap penalty is useful to penalize a start of an insertion or deletion more heavily than extending the insertion or deletion. The function Π(*C*_1_, *C*_2_) returns the color substitution score for substituting color *C*_2 _for color *C*_1_. The base and color substitutions functions are both symmetric, and are defined even if *B*_1 _= *B*_2 _for Δ, or *C*_1 _= *C*_2 _for Π. To decode an encoded sequence, we define the function Γ(*B*, *C*) that returns the decoded base using the encoded color *C *and the previous base *B *(see Figure 6). For example, to decode the encoded sequence *c *= *c*_1_,..., *c*_*n *_with a known start base *p*, we iteratively use Γ. The decoded sequence will be *x*_1 _= Γ(*p*, *c*_1_), *x*_2 _= Γ(*x*_1_, *c*_2_),..., *x*_*n *_= Γ(*x*_*n*-1_, *c*_*n*_). To encode a sequence, we define the function Φ(*B*_1_, *B*_2_) that returns a color using the bases *B*_1 _and *B*_2_, where *B*_1 _occurs before *B*_2 _in the sequence (see Figure 1). For example, to encode DNA sequence *x *= *x*_1_,..., *x*_*n*_, we assume a known start base *p *and iteratively use Φ to encode x. Here we have *c*_1 _= Φ(*p*, *x*_1_), *c*_2 _= Φ(*x*_1_, *x*_2_),..., *c*_*n *_= Φ(*x*_*n*-1_, *x*_*n*_). This encoding function is analogous to the Klein Four Group under addition or the X-OR function when the colors and DNA are represented as binary numbers [14,15,17]. The function Φ is used to encode the base sequence whereas the function Γ is used to decode the color sequence. To represent the transformation of *x *into *y*, we pair bases in *x *with bases in *y *as well as including dashes to indicate that an insertion or deletion occurred. If *x*_*i *_and *y*_*j *_are matched, then we pair *x*_*i *_and *y*_*j *_and draw: . A deletion of a base in *x *relative to *y *is represented using a dash (-) and the base *y*_*j*_, and is drawn as: . An insertion into *x *relative to *y *is represented using a dash and the base *x*_*i*_, and is drawn as: . For example, for *x *= *GATTACA *and *y *= *GATACA*, a valid alignment may be: . In this example, we apply three base substitution operators, one insertion operator, and then three base substitution operators. The base substitution operators do not change the bases in this example, but are defined for completeness when *x*_*i *_= *y*_*j*_. In this manner, we describe an alignment using the base substitution, insertion and deletion operators. To model encoding errors, we assume a two-base encoding scheme; therefore, the encoding can be visualized by placing the colors in between the bases assuming the starting base is an *A*. For the reference sequence *y*, we place colors of the encoded version of *y *in between the bases of *y*. Let *c' *be the encoded DNA sequence resulting from applying all color substitution operators to *c*. Below we place the colors of the encoded sequence *c' *between the bases of the decoded version of c'. Finally we place the original encoded sequence *c *below *c'*. Given an encoded sequence *c *= *2030311 *and target DNA sequence *y *= *GATACA *a valid alignment may be: . The placement of the color (in *y*) within the insertion (relative to *c*) is arbitrary since it is compared to the composition of the colors within insertion in *c *as will be seen later. In the above alignment, the second color is changed using a color substitution, where the second color encodes for the first and second base. Without the color substitution, the alignment would be:  illustrating the necessity to model encoding errors.

Our goal is to transform *x *into *y *by maximizing the similarity score, thus maximizing sequence similarity. In practice, *x *is an observed encoded sequence, and *y *is a decoded target or reference sequence. We prefer to penalize applications of the edit operators where base substitutions or color substitutions occur. Therefore, for all *B*_1 _≠ *B*_2 _and *C*_1 _≠ *C*_2_, we assume that Δ(*B*_1_, *B*_2_) ≤ 0, 0 ≤ Δ(*B*_1_, *B*_1_), *ε *≤ 0, *ρ *≤ 0, Π(*C*_1_, *C*_2_) ≤ 0 and 0 ≤ Π(*C*_1_, *C*_1_). Furthermore, to avoid always placing an insertion, we must have that for any *C*_1 _that *ε *+ Π(*C*_1_, *C*_1_) ≤ 0 and *ρ *+ Π(*C*_1_, *C*_1_) ≤ 0. A subtle but important point is that two adjacent color substitutions in the encoded sequence in some cases are equivalent to a base substitution in-between the two colors. An example of this equivalence can be seen in the following two sub-alignments  and . In practice we make the assumption that for any bases *B*_1_, *B*_2_, , *B*_3 _with *B*_2 _≠ , and for any colors *C*_2_, , *C*_3_,  with *C*_2 _≠  and *C*_3 _≠  such that Γ (*B*_1_, *C*_2_) = *B*_2_, Γ (*B*_2_, *C*_3_) = *B*_3_, , :

(1)

This will ensure that two adjacent color substitutions ( for *C*_2 _and  for *C*_3 _above) that are compatible with a base substitution ( for *B*_2_) will not be preferred over the compatible base substitution. Considering more complex alignments, for example whether to prefer two adjacent color substitutions or an adjacent color substitution and a base substitution, can help fine-tune the power to detect color errors as well as base substitutions by adding additional constraints on the scoring functions.

### The Algorithm

In this algorithm, we search over all possible base substitutions, base insertions, base deletions, and color substitutions. Similar to Ewans and Grant [10] and Jones and Pevzner [11], we give a recursive formula that describes the basic calculation that is repeated in our algorithm.

(2)

Intuitively, we are filling in an *n *by *m *matrix, with each cell containing 12 sub-cells. The *h *sub-cells correspond to bases that are present in *y *but deleted in *x*, the *v *sub-cells correspond to bases inserted into *x *but absent in *y*, and each *s *sub-cell represents a base *x*_*i *_(where ) aligning to a base *y*_*j *_to the reference sequence *y*. All possible color substitutions are considered by transitioning from a sub-cell , , or  to the sub-cell .

We first observe that base substitutions and color substitutions occur in tandem. This is because given the previous base *x*_*i*-1_, the subsequent base *x*_*i *_uniquely determines the joining color *c*_*i *_(or equivalently the joining color *c*_*i *_uniquely determines the subsequent base *x*_*i*_). Additionally, we assume that color substitutions do not occur directly before a base that has been deleted. In the deletion case, we have one color that spans the entire deletion. Due to base substitutions and color substitutions occurring in tandem, we must consider a color substitution while considering a base substitution, which occurs at the end of the deletion. For insertions, if the color substitution score are equal, meaning the same score is given for all color matches and color mismatches respectively, we need only consider *σ *= Γ(*φ*, *c*_*i*_) in the v-term. This reduces the number of terms over which we compute the maxima from eight terms to two terms. The simplification results from the absence of bases for which to compare the inserted base(s) as well as the observation that placing the color substitution at the end of the insertion will result in the same score as placing the color substitution anywhere else in the insertion, including the beginning of the insertion. Since base substitutions are to be penalized, as was previously assumed, we assume that the inserted bases, and therefore the colors encoding the inserted bases, are correct. Thus, when beginning or extending an insertion, we ignore the color substitution score, and consider the insertion of the base *x*_*i *_= Γ(*x*_*i*-1_, *c*_*i*_). Finally, we ignore the case where an insertion (or deletion) is directly followed by a deletion (or insertion), since for current technologies, the length of the sequences being compared are very short making this scenario (switching) very biologically unlikely. Nevertheless, to include this case requires minimal modification to Equation 2.

What is left is to describe is how to initialize , , , ,, and  for *i *> 0, j ≥ 0, and *σ *∈ {*A*, *C*, *G*, *T*}. In our specific application, we wish to align the entire encoded sequence *c *to the target sequence *y*. Therefore, we initialize for *i*>0  =  = -∞,  if *σ *= Γ (*p*, *c*_1_) and  otherwise, and for *i>1 * if *σ *= Γ (*φ*, *c*_*i*_) and  = -∞ otherwise, so that the local alignment spans the entire encoded sequence as well as allowing for an insertion at the beginning of the alignment. We initialize  = -∞ for j ≥ 0 so that the alignment does not begin with a deletion. We observe that deletions are detected on the basis that a reads spans the deletion breakpoint. This is reflected in our scoring system where we assume that a deletion has negative score, and therefore the alignment resulting from removal of a deletion at the beginning or end of the alignment has a score greater than or equal to the original alignment. We thus remove from consideration any instances of a sequence starting or ending with a deletion. We initialize  = -∞ for j ≥ 0 and *σ *∈ {*A*, *C*, *G*, *T*}. If *σ *= *p *then we t  = 0, and  = -∞ otherwise, for j ≥ 0 and *σ *∈ {*A*, *C*, *G*, *T*}. This initialization enforces that the starting base is *p*. Other initializations can find the optimal subsequence of *x *that aligns to *y*, among other applications [10,11]. To find the optimal local alignment we search over cells  and  for a cell with maximum score, again ignoring the case where the alignment ends with a deletion, and backtrack to recover a maximum scoring alignment.

From Equation 2, and for each *i *and *j*, we must calculate maxima over 88 different values, which can be reduced to 64 values if the color match and color mismatch scores respectively are the same. In contrast, the Dynamic Programming solution with affine gap penalties to compare sequences with no encoding requires the calculation of a maxima over 7 different values [10,11]. Although the running time of this algorithm is O(*nm*), where *n *is the length of the encoded sequence and *m *is the length of the target sequence, the running time is nonetheless greater than the algorithm without encoding as seen in practice (see Results).

### Simulations

To evaluate the power of the algorithm, we created sets of 100,000 test sequences randomly sampled from the Human genome (build 36), and gave each a known number of errors, base substitutions, insertions and deletions. For encoded sequences, we model errors as color substitutions (encoding errors) and for decoded sequences we model errors as base substitutions. It is possible for a class of alignments to have equal likelihood, and therefore we define an alignment to be correct if the alignment returned has equal score to the true alignment. To evaluate the performance of the algorithm, we created 1,000,000 artificial sequences from the Human genome (build 36) with no edits applied. In both cases, we evaluated sequences of length 25 and 50, reflecting a range of possible and currently available sequences generated with color space encoding. The target DNA reference sequence had length three times the length of the encoded sequence to allow for potential insertions and deletions to be placed correctly. For the simulations, in accordance with Equation 1, we set *ρ *= -175, *ε *= -50, Π(*C*_1_, *C*_2_) = -125 (*C*_1 _≠ *C*_2_), Π(*C*_1_, *C*_1_) = 0, Δ(*B*_1_, *B*_2_) = -150 (*B*_1 _≠ *B*_2_), and Δ(*B*_1_, *B*_1_) = 50. Since the color match and color mismatch scores respectively are the same, we are able to make the simplification to the v-term in Equation 2 as described above. For these evaluations, we used a dual quad-core Intel Xeon E5420 machine at 2.5 GHz, with 32 GB of RAM and 2TB of RAID 0 disk space, although the actual hardware requirements of the algorithm itself are negligible relative to any modern computer. The implementation for all the simulations performed can be found in BFAST at , which was configured using the –enable-unoptimized-sw argument (Homer N, Merriman B, Nelson SF: BFAST: the BLAT-like Fast Accurate Search Tool for Large-Scale Genome Resequencing, submitted).

## Authors' contributions

NH conceived of and implemented the algorithm, and performed the analyses. BM, and SFN advised on the development and analysis of the method, and producing the manuscript.
